# Advanced intermediate temperature sodium–nickel chloride batteries with ultra-high energy density

**DOI:** 10.1038/ncomms10683

**Published:** 2016-02-11

**Authors:** Guosheng Li, Xiaochuan Lu, Jin Y. Kim, Kerry D. Meinhardt, Hee Jung Chang, Nathan L. Canfield, Vincent L. Sprenkle

**Affiliations:** 1 Electrochemical Materials and Systems Group, Energy Processes and Materials Division, Pacific Northwest National Laboratory, Richland, 99352 Washington, USA

## Abstract

Sodium-metal halide batteries have been considered as one of the more attractive technologies for stationary electrical energy storage, however, they are not used for broader applications despite their relatively well-known redox system. One of the roadblocks hindering market penetration is the high-operating temperature. Here we demonstrate that planar sodium–nickel chloride batteries can be operated at an intermediate temperature of 190 °C with ultra-high energy density. A specific energy density of 350 Wh kg^−1^, higher than that of conventional tubular sodium–nickel chloride batteries (280 °C), is obtained for planar sodium–nickel chloride batteries operated at 190 °C over a long-term cell test (1,000 cycles), and it attributed to the slower particle growth of the cathode materials at the lower operating temperature. Results reported here demonstrate that planar sodium–nickel chloride batteries operated at an intermediate temperature could greatly benefit this traditional energy storage technology by improving battery energy density, cycle life and reducing material costs.

## Abstract (web summary)

Sodium metal halide batteries are attractive technologies for stationary electrical energy storage. Here, the authors report that planar sodium-nickel chloride batteries operated at an intermediate temperature of 190 °C display larger energy densities than tubular batteries operated at higher temperatures.

## Graphical abstract

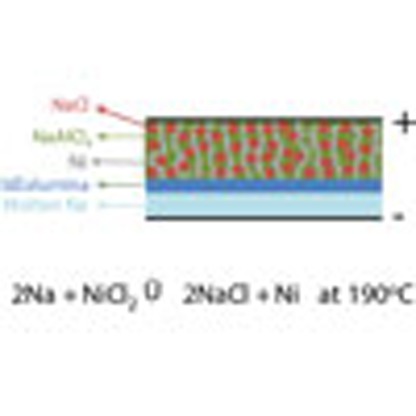

Recently, molten-sodium (Na) beta-alumina batteries have been considered as one of the most attractive stationary electric energy storage systems, which are crucial to stimulate the growth of renewable energy resources and to improve the reliability of electric power grids^1^,^2^,^3^,^4^,^5^. Sodium–sulfur (Na–S)^6^ and sodium-metal halide batteries (ZEBRA)^7^ are two typical molten-Na beta-alumina batteries; however, recent fire incidents involving Na–S battery systems have increased general concern about the application of Na–S batteries as stationary energy storage devices. Although they share some features (for example, molten-sodium and β″-alumina solid electrolyte) in common with Na–S batteries, ZEBRA batteries can provide several advantages over Na–S batteries, including superior battery safety, high open-circuit voltage, lower operating temperature and ease of assembly in the discharged state without using metallic sodium in the anode^2^,^4^,^7^.

Among various ZEBRA battery redox chemistries^8^,^9^,^10^,^11^,^12^, the sodium–nickel chloride (Na–NiCl_2_) battery has been most widely investigated in the past^13^,^14^,^15^. The overall redox reaction of a Na–NiCl_2_ battery during charging and discharging processes is described as follows:


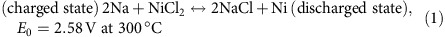


Despite the relatively simple redox reaction, cell degradation mechanisms of Na–NiCl_2_ batteries have not been clearly understood in the past. In our recent studies^16^, we have reported detailed correlations between NaCl/Ni particle growth (Ostwald ripening) and battery-operating conditions, such as C-rate, cathode formula and cycling capacity window. The main parameters that lead to faster Ni particle growth are higher current density, state of charge (SOC) at end of charge (EOC) and Ni/NaCl ratio. In the case of NaCl, significant growth has a close correlation with the cycling capacity window^16^. To achieve sustainable battery cycle life, the conventional tubular Na–NiCl_2_ batteries are loaded with excessive Ni content in the cathode and also are operated with a shallow capacity window. The theoretical specific capacity and energy density of Na–NiCl_2_ ZEBRA batteries obtained from reaction (1) are 305 mAh g^−1^ (without considering the melt) and 788 Wh kg^−1^ (open-circuit voltage at 2.58 V), see Supplementary Table 1. Despite the quite impressive theoretical energy density of Na–NiCl_2_ batteries, general energy density obtained from a conventional tubular Na–NiCl_2_ battery (operated at ca. 300 °C) is about 95–120 Wh kg^−1^ due to excessive Ni content and shallow capacity window^2^,^3^,^5^. Detailed plots of energy density versus Ni content with different cycling windows are shown in Fig. 1 (see Supplementary Fig. 1 as well). Resolving this shortcoming on a material and cost level requires creating new platforms based on innovative scientific and technical approaches. Excited by the magnitude and implications of revisiting Na–NiCl_2_ ZEBRA battery technology, the research and industrial communities are seeking a revolutionary breakthrough that could enable substantially lower cost for materials and operations, as well as superior battery cycle life and safety.

In our previous work^17^, we found that the operating temperature has significant influence on the cell chemistries during the battery cycling. The cell polarization, an important indicator of cell degradation, was found to increase faster at 280 °C than at 175 °C due to faster grain growth in the cathode ingredients. From a cell-operation point of view, lower temperature can potentially reduce costs associated with cell packing and reduce heat loss. In a recent report, Gerovasili *et al*.^18^ concluded that lower heat transfer losses at 240 °C could result in up to 49% reduction in heating energy compared with operation at 275 °C. It should be noted that it is intrinsically difficult for a tubular ZEBRA battery to operate below 240 °C.

Drawing inspiration from the temperature-dependent particle growth, we construct a planar intermediate temperature (IT) Na–NiCl_2_ ZEBRA battery technology, which allows the cells to be operated at an IT of 190 °C with considerable discharge power as high as 75 mW cm^−2^ (∼0.6 C). Extensive investigations of cell performance and fundamental understanding of cathode degradation mechanisms at 190 °C are studied, and indicate that this novel planar IT Na–NiCl_2_ ZEBRA battery technology could have a much higher specific energy density (350 Wh kg^−1^) and much more stable cycle life than the state-of-the-art ZEBRA battery.

## Results

### Battery performances of planar IT ZEBRA battery

**Figure 2:**
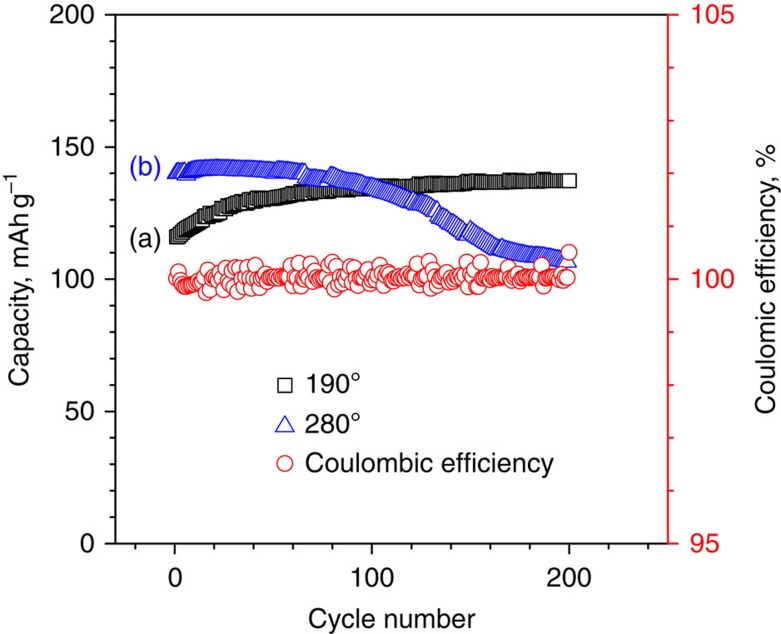
Capacity retention and Coulombic efficiency plots. Na–NiCl_2_ cells were operated at two different temperatures: (a) 190 (black) and (b) 280 °C (blue). Coulombic efficiency is shown in red. Cells were charged with a constant current (7 mA cm^−2^, ∼C/7) and were discharged with a constant power (25 mW cm^−2^, ∼10 mA cm^−2^, ∼C/5).

We constructed a planar IT ZEBRA battery, using a β″-alumina solid-state electrolyte (BASE; 3 cm^2^ effective areas), a Ni/NaCl granule cathode (157 mAh, 52.3 mAh cm^−2^) and NaAlCl_4_ as a secondary electrolyte. Detailed information can be found in the experimental section. All batteries were charged and discharged between 2.8 V (EOC) and 2.0 V (end of discharge) to prevent side reactions occurring due to over-charging and over-discharging. Figure 2 shows battery performance for cells operated at two different temperatures (190 and 280 °C) with a constant discharge power of 25 mW cm^−2^ (∼10 mA cm^−2^, ∼C/5). An initial capacity of 106 mAh g^−1^ was observed for IT Na–NiCl_2_ batteries tested at 190 °C. Interestingly, IT Na–NiCl_2_ batteries (Fig. 2, black) showed a capacity increase for the first 100 cycles and then stabilized with a capacity of 137 mAh g^−1^ at the 200th cycle. The specific discharge energy density for IT Na–NiCl_2_ batteries was 340 Wh kg^−1^ (200th cycle), which is by far the highest energy density demonstrated for Na–NiCl_2_ batteries to the best of our knowledge. Identical batteries tested at 280 °C are also shown in Fig. 2 (blue). The initial capacity of 140 mAh g^−1^ was obtained for the battery operated at 280 °C. The higher initial capacity obtained at 280 °C compared with that at 190 °C is likely due to better sodium wetting on the BASE at 280 °C. In contrast with IT Na–NiCl_2_ batteries at 190 °C, the capacity of batteries operated at 280 °C decreased drastically, to 107 mAh g^−1^ (76% retention) over 200 cycles. The more stable performance of the Na–NiCl_2_ batteries at 190 °C than at 280 °C indicates that the lower operation temperature could be the most critical factor for obtaining sustainable cell performance, which has been surprisingly understated in the past. As shown in Supplementary Fig. 2, cells have been also tested with a higher discharge power (75 mW cm^−2^, ∼30 mA cm^−2^, ∼0.6 C) at 190 and 280 °C. Similar to results shown in Fig. 2, the capacity was more stable for batteries operated at 190 °C than at 280 °C. The Coulombic efficiencies shown in Fig. 2 (red) and Supplementary Fig. 2 (red) of all tested batteries are nearly 100%, which is due to use of BASE as the sodium-ion conducting solid-state electrolyte.

**Figure 3:**
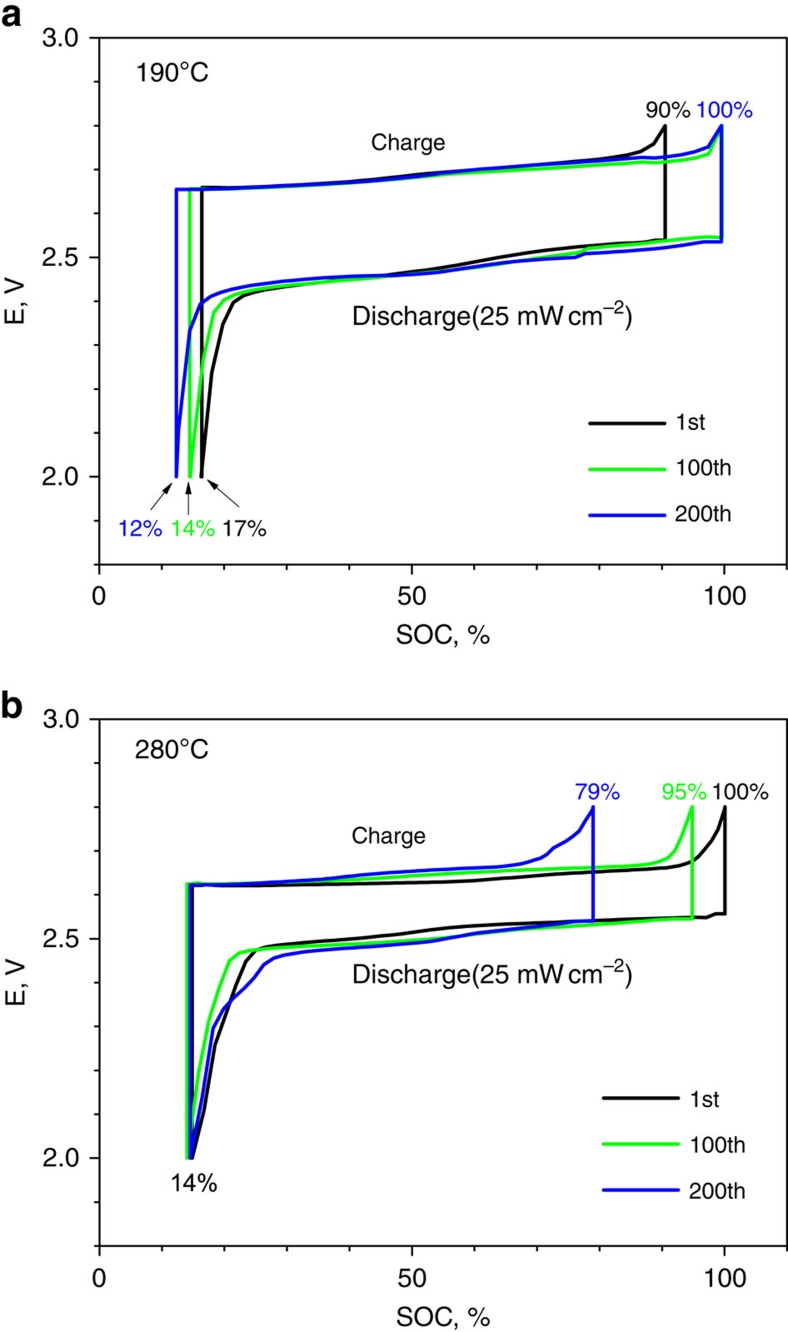
Voltage profiles for planar IT Na–NiCl_2_ batteries. Cells were operated with constant-current charge (7 mA cm^−2^, ∼C/7) and constant-power discharge (25 mW cm^−2^, 10 mA cm^−2^, ∼C/5). Voltage profiles versus SOC are shown for 1st (black), 100th (green) and 200th (blue) cycles at two different temperatures (**a**) 190 and (**b**) 280°C, respectively.

To further understand the effect of temperature on cell performance, voltage profiles (1st, 100th and 200th cycles) versus SOC for cells tested at 190 and 280 °C are shown in Fig. 3 (25 mW cm^−2^) and Supplementary Fig. 3 (75 mW cm^−2^). For the cells operated at 190 °C (Fig. 3a), SOC at the EOC (SOC_EOC_) and SOC at the end of discharge (SOC_EOD_) for the 1st cycle were determined to be 90 and 17%, respectively. In further battery cycles, SOC_EOC_ was gradually increased to 100% and SOC_EOD_ was decreased to 12%. These adjustments are responsible for the progressively increasing capacity for the cells operated at 190 °C (Fig. 2), since the capacity of a battery can be calculated as follows:


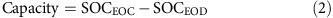


For instance, capacities of the 1st and 200th cycles for the cells operated at 190 °C were 73 and 88%, respectively, as calculated using equation (2). In contrast with the lowest capacity having been observed in the early stage of tested cycles at 190 °C, the SOC_EOC_ and SOC_EOD_ for the 1st cycle at 280 °C were 100 and 14%, respectively, which resulted in the largest initial capacity of 86% as shown in Fig. 3b. The higher initial capacity observed at the operating temperature of 280 °C than at 190 °C is most likely due to better sodium wetting on the BASE at the higher operating temperature. However, the SOC_EOC_ of cells operated at 280 °C rapidly decreased to 79% after the 200th cycle, which results in a capacity of 65% (76% capacity retention). On the other hand, a quite stable SOC_EOD_ observed over 200 cycles indicates that degradation on the anode side (sodium wetting) is negligible for 280 °C as shown in Fig. 3b. The rapid degradation of capacity for the cells operated at 280 °C is more likely due to cathode degradation at the higher operating temperature.

### Scanning electron microscopy of cathode materials

**Figure 4:**
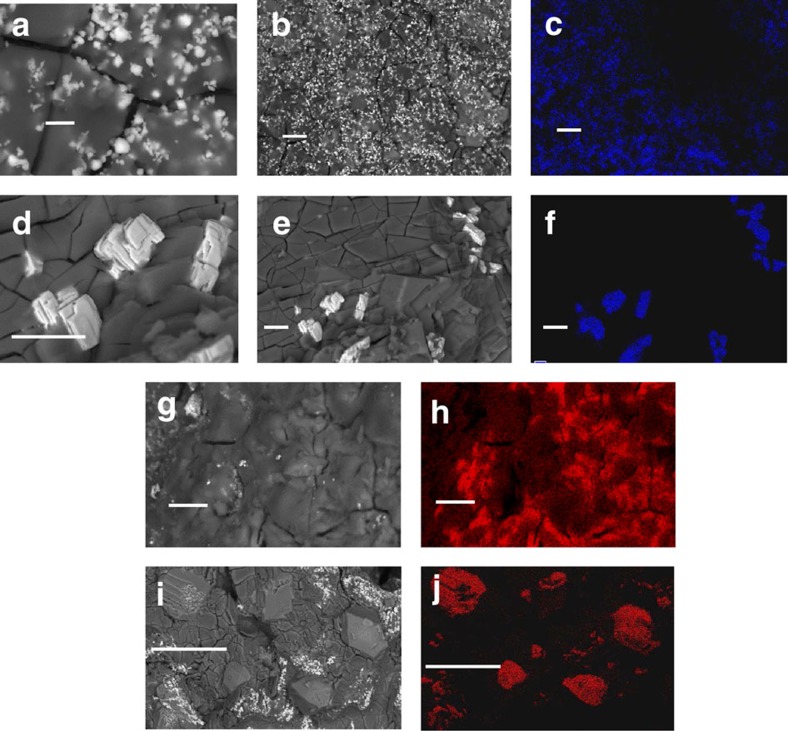
SEM images for cathode materials. Cathode materials were retrieved from cells operated for 200 cycles. Ni particles are shown for cells operated at 190 °C (25 mW cm^−2^) at (**a**) × 7,500 (scale bar, 2 μm), (**b**) × 1,000 (scale bar, 10 μm) and (**c**) Ni mapping × 1,000 (scale bar, 10 μm). Ni particles from cells operated at 280 °C (25 mW cm^−2^) are shown at (**d**) × 3,000 (scale bar, 10 μm), (**e**) × 1,000 (scale bar, 10 μm) and (**f**) Ni mapping × 1,000 (scale bar, 10 μm). NaCl particles are shown for cells operated at 190 °C (25 mW cm^−2^) at (**g**) × 2,000 (scale bar, 10 μm) and (**h**) Na mapping × 2,000 (scale bar, 10 μm); (**i**) × 300 (scale bar, 100 μm) and (**j**) Na mapping × 300 (scale bar, 100 μm) for 280 °C.

To investigate the correlation between morphology changes in Ni/NaCl cathodes and cell performance, the cells operated with a constant discharge power of 25 mW cm^−2^ were disassembled after 200 cycles and the fracture surfaces were examined using scanning electron microscopy (SEM)/energy-dispersive x-ray spectrometry (EDS). For cathodes retrieved from cells tested at 190 °C, lighter spots shown in Fig. 4a (high resolution) and Fig. 4b represent Ni particles and corresponding images of Ni mapping are shown in Fig. 4c. Similarly, Nickel particles are shown in Fig. 4d (high resolution) and Fig. 4e,f (Ni mapping) for cells tested at 280 °C. Quite different sizes of Ni particles were observed for the cells operated at 190 °C compared with those at 280 °C. The typical particle size of Ni cathodes for the cells tested at 190 °C was 1–2 μm (Fig. 4a,b), which is similar to the initial particle size of raw Ni powders. However, significant Ni particle growth, up to 10 μm (Fig. 4d,e), was observed for the cells tested at 280 °C. Particle sizes of NaCl were also determined from SEM images (Fig. 4g,i) and corresponding images of Na mapping (Fig. 4h,j). NaCl particle sizes in the cells operated at 190 and 280 °C were ∼5 μm and ∼50 μm, respectively. SEM/EDS measurements were also performed for the cells operated with a higher discharge power of 75 mW cm^−2^ at 190 and 280 °C. Similar to the cells tested at a discharge power of 25 mW cm^−2^, larger NaCl and Ni particles were observed in cells operated at 280 °C than in cells operated at 190 °C with a discharge power of 75 mW cm^−2^, as shown in Supplementary Fig. 4.

### Ni and NaCl particle growth

The sizes of Ni and NaCl particles from tested cells, cell cycling conditions and cell performances are summarized in Table 1 for a better comparison. It is quite clear that the particle sizes of Ni and NaCl for the tested cells show a strong dependence on the cell-operating temperature. For instance, the average Ni particle size at 280 °C is around 10 μm, which is significantly larger than the average particle size of 1–2 μm observed at 190 °C. Similarly, the NaCl particle size at 280 °C was 50 μm, which is much larger than an average particle size of 5–10 μm at 190 °C. Considering the particle size of raw Ni powders (1–2 μm) and NaCl powders (∼5 μm), the particle growth at 190 °C is much slower than that at 280 °C. The morphology evolution of cathode materials has been considered as the most important cause of the degradation of Na–NiCl_2_ batteries. For the charging process, the main reactions in the cathode side of Na–NiCl_2_ batteries are the dissolution of NaCl particles into the melt and the formation of NiCl_2_ layers on the surfaces of Ni particles. Larger particles of active ingredients existing in the cathode will lead to a sluggish dissolution of NaCl and less surface area of Ni particles, which will eventually cause a limited charging capacity. This is in good agreement with the observations shown in Table 1. For example, an IT Na–NiCl_2_ battery operated at 190 °C can still be charged to 100% SOC after 200 cycles due to the minimal morphology changes in the cathode, but identical cells operated at 280 °C can be only charged up to 79% SOC after 200 cycles due to the accelerated particle growth in the cathode.

The mechanism of particle growth in Na–NiCl_2_ batteries operated at 280 °C has been proposed in our previous study by attributing it to Ostwald ripening^16^. Here we would like to extend the particle growth mechanism by including the operating temperature as an important factor. In the literature^19^,^20^, it has been generally understood that the influence of temperature on Ostwald ripening is through its effects on various parameters, such as the equilibrium solubility, the diffusion-influenced growth coefficient, the phase-transition energy and interfacial energy. Here, temperature effects on equilibrium solubility and diffusion-influenced growth coefficient are particularly important to understanding the enhanced particle growth in Na–NiCl_2_ batteries at the higher temperature. For instance, the solubility of cathode materials (NaCl and NiCl_2_) will drastically decrease with a decrease in the operating temperature^21^. The diffusion coefficients of dissolved NaCl and NiCl_2_ will decrease as well, due to the increased viscosity of the melt. Since the equilibrium solubility and diffusion-influenced growth coefficient are proportional to the solubility and diffusion coefficient, respectively, particle growth by Ostwald ripening will be greatly suppressed at lower operating temperatures.

## Discussion

**Figure 5:**
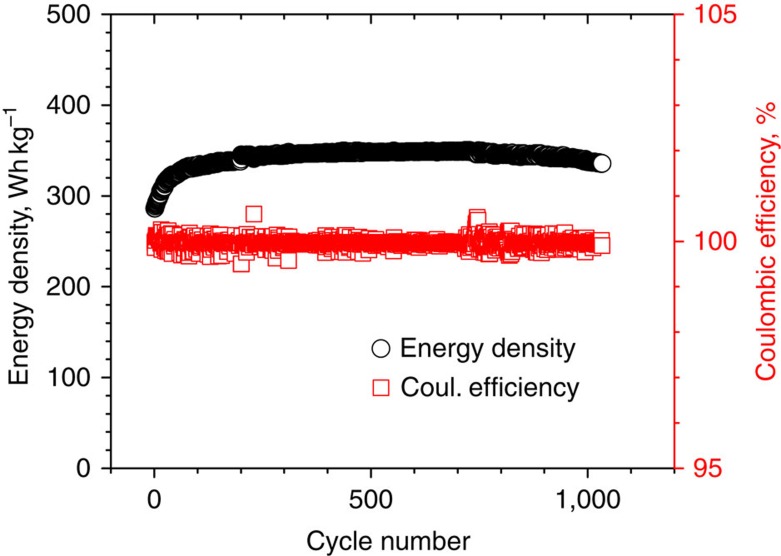
Energy density and Coulombic efficiency. Planar IT Na–NiCl_2_ cells were operated at 190 °C over 1,000 cycles in a period of 1 year and 6 months. No degradation was observed for the first 700 cycles. A degradation rate of <0.01% per cycle was obtained for the test after the 700th cycle.

For stationary energy storage, long-battery lifetime and lower materials cost are two critical factors. As shown in Fig. 5, long-term cycling of an IT Na–NiCl_2_ battery showed an excellent stability over 1,000 cycles, which was a testing period of 1 year and 6 months. No battery degradation was observed until the 700th cycle, and the degradation rate thereafter was <0.01% per cycle. The energy density of an IT Na–NiCl_2_ battery shown in Fig. 5 was generally over 330 Wh kg^−1^. Because of the ultra-high energy density of the IT Na–NiCl_2_ batteries demonstrated in this work, the materials cost of cathodes (notably the cost of Ni) can be greatly reduced compared with the conventional tubular Na–NiCl_2_ batteries, which would be considered as a significant saving. One concern with decreasing the operational temperature from 280 °C to an IT of 190 °C is the inevitable loss in energy efficiency of a Na–NiCl_2_ battery. Cells operated at the lower temperature showed generally larger overpotentials than cells operated at the higher temperature as shown in Fig. 3 and Supplementary Fig. 3. Overall energy efficiency plots are shown in Supplementary Fig. 5. Typical overall energy efficiencies at the 200th cycle are 91.6% (190 °C, 25 mW cm^−2^), 86.5% (190 °C, 75 mW cm^−2^), 92.3% (280 °C, 25 mW cm^−2^) and 88.4% (280 °C, 75 mW cm^−2^). The energy efficiencies of cells operated at 190 °C show about 1% decrease in overall efficiencies versus those operated at 280 °C; this is due to the close relationship between the operating temperature, interfacial resistance and sodium-ion conductivity in the cell. However, the decrease in energy efficiency is trivial compared with the advantages of the lower operating temperature.

In conclusion, we have developed a novel planar Na–NiCl_2_ battery that can be operated at an IT of 190 °C. This planar IT Na–NiCl_2_ technology was able to deliver an ultra-high energy density (350 Wh kg^−1^) with very long cycle life (over 1,000 cycles) and excellent capacity retention (no decay until the 700th cycle, 0.01% per cycle thereafter). This work accomplished a breakthrough towards making Na–NiCl_2_ battery technology more competitive for stationary energy storage applications.

## Methods

### Material preparation and battery assembly

Detailed methods for preparing the Ni/NaCl cathodes and planar cell assembly procedures have been reported previously^16^. Cathode materials in the discharged state consisted of 62.2 wt% Ni (Novamet, Type 255), 34.2 wt% NaCl (Alfa Aesar, 99.99%) and 3.6 wt% additives (1.6 wt% of FeS, 0.6 wt% of aluminium powder, 0.5 wt% of NaI and 0.9 wt% NaF). The cathode materials were thoroughly mixed using a low-energy ball-milling method. Then, well-mixed cathode powders were transferred into a granulator (Freund TF-Labo) to make cathode granules. NaAlCl_4_, the secondary electrolyte, was synthesized following our previously reported method^22^. The ratio of NaAlCl_4_ and Ni/NaCl cathode is 0.5:1. The specific capacity of the button cell in this work was 157 mAh g^−1^ for without considering and 105 mAh g^−1^ with including the mass of the melt (NaAlCl_4_). The button cell consisted of battery cases (stainless steel) for cathode (attached with a thin molybdenum film liner) and anode sides, a molybdenum cathode current collector, a stainless steel anode current collector, an α-alumina (99.5% purity) fixture and a planar composite yttria-stabilized zirconia (YSZ)/ BASE (3 cm^2^ active area) disc. A schematic view of the planar design of the cell used in this work is shown in Supplementary Fig. 7. BASE discs with 500-μm thickness were fabricated using the vapour–phase-conversion process^7^,^11^, which is briefly described as follows, 70% of α-alumina (α-Al_2_O_3_, Almatis A16-SG, >99%) powder and YSZ powder (8YSZ, Imerys Fused Minerals, 5.4 wt% Y_2_O_3_) were first mixed to the desired volume ratios (7:3), and followed by attrition milling in iso-propanol to achieve a median particle size (d_50_) <0.5 μm. This not only ensures nearly uniform particle size, but also eliminates regions of compositional non-uniformity. Slurries were prepared by mixing attrition milled powders with solvents made of methyl ethyl ketone/ethanol (4:1), dispersant (PS-131, AkzoNobel, 0.5 wt%), a binder (polyvinylbutyral, B-79, 4.38 wt%) and a plasticizer (benzyl butyl phathalate, Alfa Aesar, 98%, 4.73 wt%) and then tape cast to a thin sheet (125 μm). Four layers of thin sheets were stacked and run through a hot roll laminator at 135 °C to create a monolithic laminate. The laminated sheet was laser cut to a desired size (3 cm^2^) and sintered under a programed thermal process (0.5 °C min^−1^ to 190 °C for 2 h, 0.5 °C min^−1^ to 400 °C for 1 h and 3°C min^−1^ to 1,500°C for 2 h) to allow removing solvents, burn-out of the binder and sintering. After sintering, the α-Al_2_O_3_/YSZ parts were placed in ‘packing powder' of β″-Al_2_O_3_ powder with 10 wt% NaAlO_2_ and heat treated at 1,400 °C for 10 h. The packing powder acts as the sodium source to convert the sintered α-Al_2_O_3_ to β″-Al_2_O_3_. The results of ionic conductivity measurement and SEM cross-section for BASEs used in this work are shown in Supplementary Fig. 6. The activation energy obtained from Arrhenius plot is about 0.24 ev. The cell assembly process can be briefly described as follows. First, a BASE disc was glass-sealed to an α-alumina fixture, and the anode side of the BASE disc was pretreated with an aqueous lead acetate (Pb[CH_3_COO]_2_) solution. Then, the fixture was heat treated at 400 °C in an inert environment to form a thin layer of Pb/PbO on the BASE surface facing the anode. It has been reported that this thin layer of Pb/PbO improves initial sodium wetting on the surface of the BASEs^16^,^23^. The fixture was then transferred into a nitrogen-purged glove box for the cell assembly. In all, 1 g of cathode granules was added to cathode side of the fixture and 0.5 g of melt was vacuum infiltrated into the granules at an elevated temperature of 200 °C. A small amount of metallic sodium (<5 mg) was added to the anode shim (an initial contact) before the fixture was enclosed with cell cases by the compression sealing using metal O-rings.

### Battery tests

Assembled cells were transferred from the glove box into furnaces for battery cycle tests in air. The cells were tested at two different temperatures, 190 and 280 °C, and the temperatures of the cells were closely monitored by K-type thermocouple wire gauges attached to the cell cases. Galvanostatic tests were performed using an Arbin potentiostat (MSTAT 8000). The cell test consisted of conditional and regular cycles. During the conditional cycles, the first charging process of the cells is conducted in three stages with different charging currents (0.6 mA for 2 h, 1 mA for 10 h and 10 mA thereafter) until the cell voltage reaches 2.8 V. For the first discharge, the cells are discharged at a constant current (10 mA, C/15) until the cell voltage decreases to 1.8 V. After the first cycle is completed, the cells are cycled between 1.8 V (discharge limit) and 2.8 V (charge limit) with a constant current (10 mA, C/15) for two additional cycles. Regular cycle tests were conducted on the cells charged with the conditional cycle. During the regular cycle test, the cells were cycled between 2.0 V (discharge limit) and 2.8 V (charge limit). A constant charge current of 20 mA (∼C/7) and a constant discharge power of 25 mW cm^−2^ (∼C/5, 10 mA cm^−2^) or 75 mW cm^−2^ (∼0.6 C, 30 mA cm^−2^) were applied for the charging and discharging steps, respectively.

### Scanning electron microscopy

SEM characterization on the fracture surfaces of the cathodes was done on JEOL JSM-5900LV equipped with an Oxford EDS with lithium drift detector and JEOL JSM-7001F (field emission) equipped with an Oxford EDS system with silicon drift detector. Batteries were disassembled in a glove box and the desired cathode materials were delivered to the SEM chamber with minimum exposure to the air. The particle sizes of Ni in the cathodes were determined from SEM backscattering images combined with elemental mapping of Ni. Similar to Ni analysis, the particle sizes of NaCl in the cathodes were determined from SEM backscattering images combined with elemental mapping of Na.

## Additional information

**How to cite this article:** Li, G. *et al*. Advanced intermediate temperature sodium–nickel chloride batteries with ultra-high energy density. *Nat. Commun.* 7:10683 doi: 10.1038/ncomms10683 (2016).

## Supplementary Material

Supplementary Information Supplementary Figures 1-7 and Supplementary Table 1

## Figures and Tables

**Figure 1:**
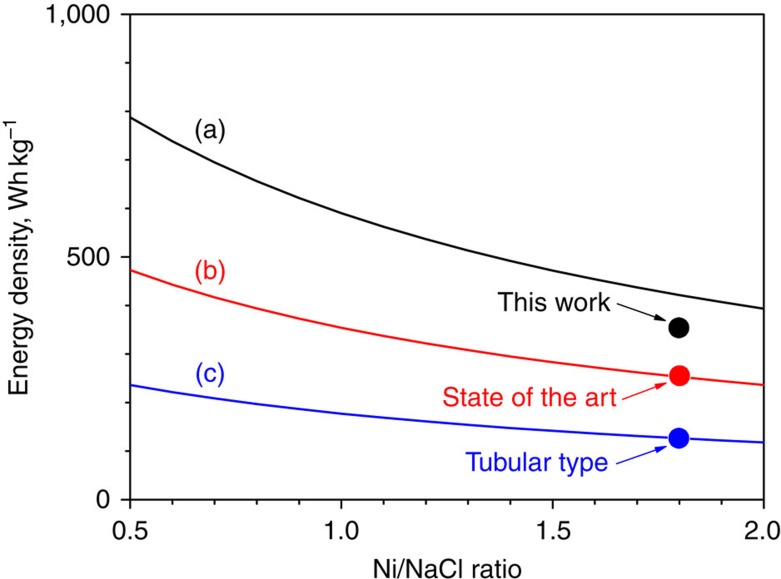
Specific energy density. Specific energy densities were calculated on different cycling capacity windows for (a) 100, (b) 60 and (c) 30%. Energy density was calculated without considering the weight of melt required in the cathode. See Supplementary Fig. 1 for the specific energy density plots calculated with the weight of melt included.

**Table 1:** Grain sizes of Ni and NaCl particles and other battery performance data for cells cycled at different temperatures and discharge powers.

	190 °C		280 °C	
Discharge (mW cm^−1^)	25	75	25	75
C-rate	C/5	0.6 C	C/5	0.6 C
Ni (μm)	1–2	1–2	∼10	∼10
NaCl (μm)	<5	∼10	∼50	∼50
EOC (%)†	100	100	79	78
EOD (%)†	12	17	14	20
Capacity window (%)	88	83	65	58
Energy density (Wh g^−1^)	340	300	258	220
Degradation (%)	19*	13*	−24	−30

EOC, end of charge; EOD, end of discharge.

^*^Positive degradation is due to the increased capacity of the 200th versus the 1st cycle at 190 °C.

^†^EOC and EOD of 200th cycle are shown in the table.
