# Supplementary material for: Advanced intermediate temperature sodium–nickel chloride batteries with ultra-high energy density
Source: Nat Commun. 2016 Feb 11;7:10683. doi: 10.1038/ncomms10683 (PMC4753253; doi:10.1038/ncomms10683)
Supplement: Supplementary Information — Supplementary Figures 1-7 and Supplementary Table 1 [file ncomms10683-s1.pdf]

Supplementary Information

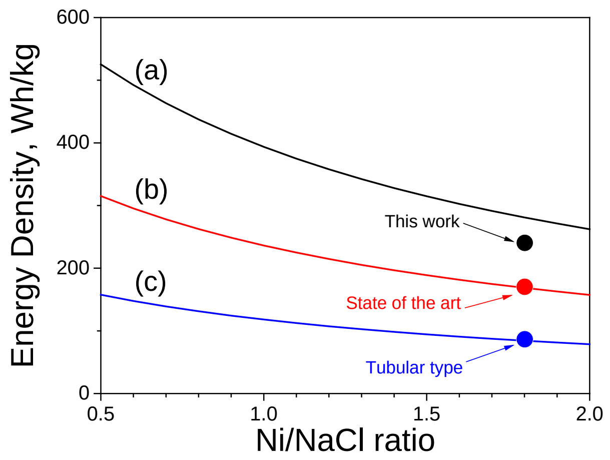

Supplementary Figure 1. Specific energy density vs. Ni/NaCl ratio with the weight of the melt included for different capacity windows of (a)100%, (b) 60%, and (c) 30%. The weight ratio (1:0.5) between the NaCl/Ni cathode and the melt was used for calculating specific energy density.

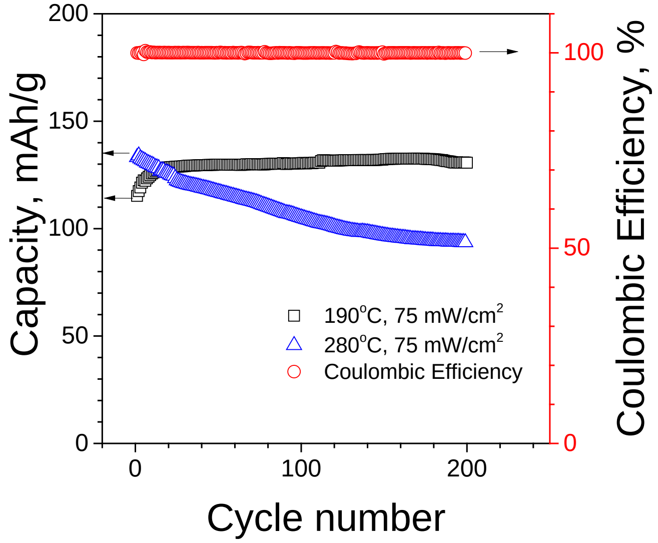

Supplementary Figure 2. Capacity retention and coulombic efficiency plots of Na-$NiCl_2$ cells operated at two different temperatures: (a) 190°C and (b) 280°C. Cells were charged with a constant current (7 mA/cm^2, ~C/7) and were discharged with a constant power (75 mW/cm^2, ~0.6C).

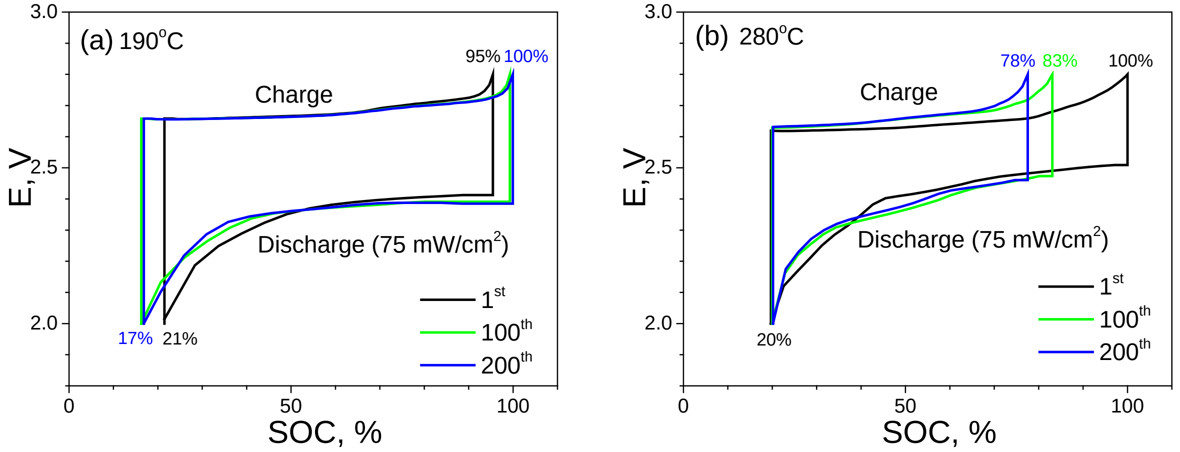

Supplementary Figure 3. Voltage profiles (vs. SOC) for planar Na-$NiCl_2$ ZEBRA batteries with constant-current charge (7 mA/cm^2, ~C/7) and constant-power discharge (75 mW/cm^2, ~0.6C) at 1st, 100th and 200th cycle: (a) 190°C and (b) 280°C.

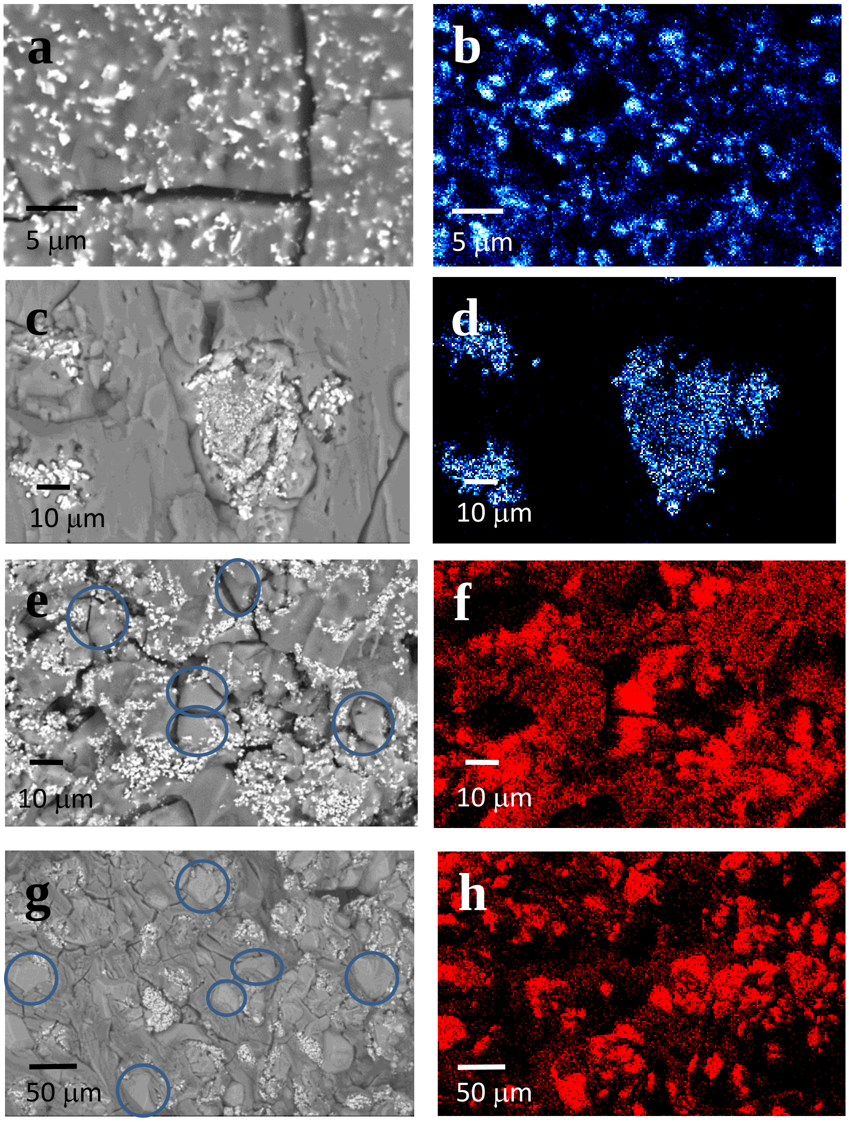

Supplementary Figure 4. SEM images for cycled cathode materials retrieved from cells operated after 200 cycles: (a) 190°C, 75 mW/cm^2, 3000×; (c) 280°C, 75 mW/cm^2, 1000×; (e) 190°C, 75 mW/cm^2, 1000×; (g) 280°C, 75 mW/cm^2, 300×. Images (b) and (d) are Ni mapping for (a) and (c), respectively, and (f) and (h) are Na mapping for (e) and (g), respectively.

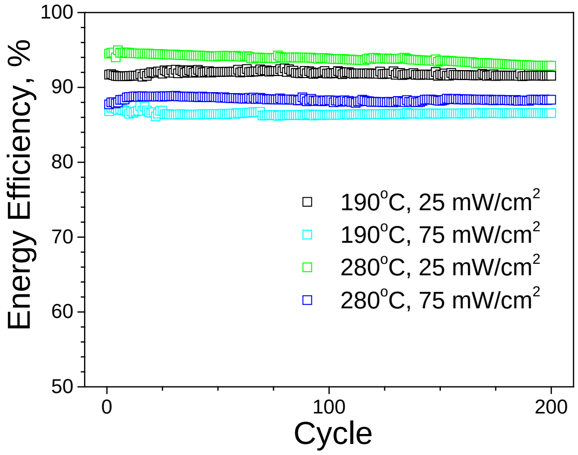

Supplementary Figure 5. Energy efficiencies of Na-$NiCl_2$ ZEBRA cells tested in this work.

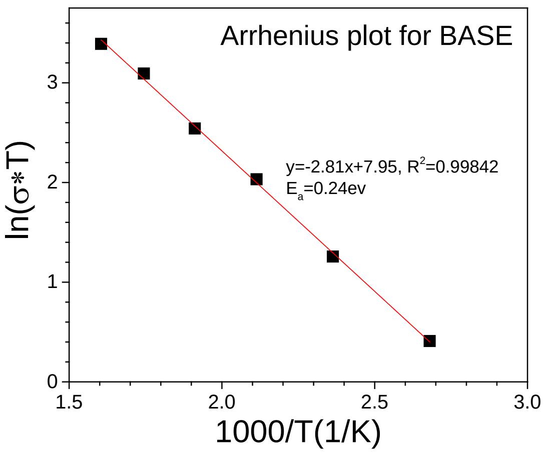

(a)

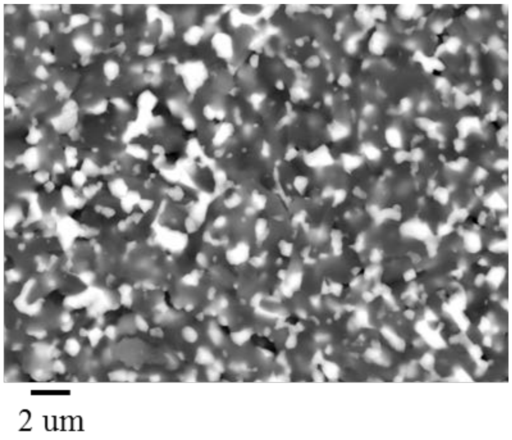

(b)

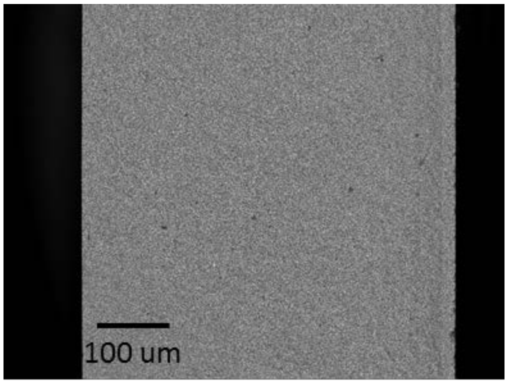

(c)

Supplementary Figure 6. Arrhenius plot of the conductivities (a) and high and low magnification (b) SEM cross section (c) for BASE used in this work.

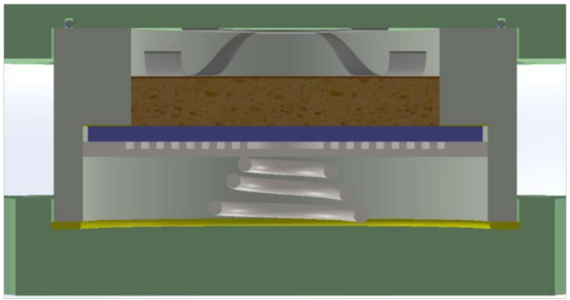

(a)

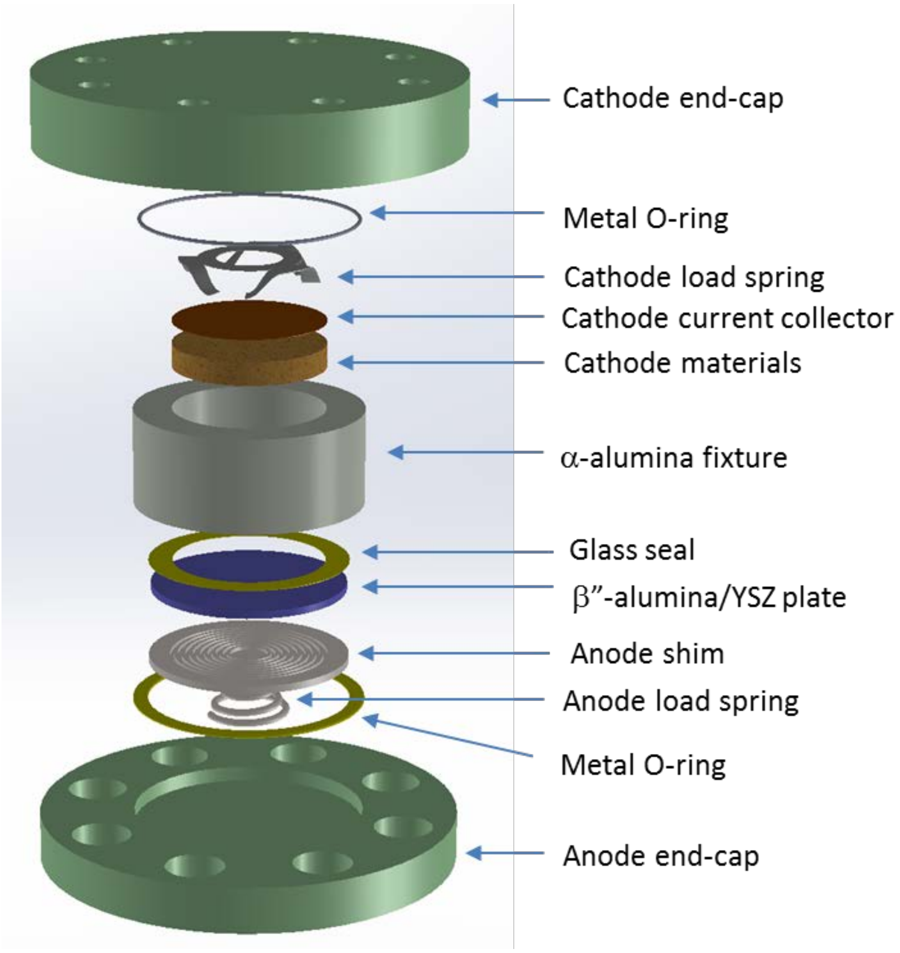

(b)

Supplementary Figure 7. (a) Schematic view of an assembled planar Na-$NiCl_2$ cell. (b) Expanded view of a planar Na-$NiCl_2$ cell design.

Supplementary Table 1. Specific capacity and energy density for Na-$NiCl_2$ battery.

Ni/NaCl molar ratio	NaCl (g)	Ni (g)	Additives (g)	Cathode weight (g)	Cell Capacity (mAh)	Specific Capacity (mAh/g)	Specific Energy* (Wh/kg)
0.5 (Stoich.)	0.666	0.334	0	1	305	305	787
1.8 (This work & tubular cell)	0.342	0.622	0.036	1	157	157	405

*E=2.58 V for output voltage.
